# Addition of telitacicept to glucocorticoid reduces abdominal pain duration and relapse rate in children with severe abdominal Henoch-Schönlein purpura: a single-center retrospective matched cohort study

**DOI:** 10.3389/fimmu.2026.1865753

**Published:** 2026-09-16

**Authors:** Xuemei Jiang, Hongye Wang, Mifeng Yang, Xiran Yang, Yabin Liao, Jia Zhang, Jiyu Chen, Helin Yan, Yingyi Zhang, Mengqian Zhouyang, Yan Gu, YangCui Wei, Bo Zhao

**Affiliations:** Nephrology, Rheumatology and Immunology Department, Kunming Children’s Hospital, Yunnan Provincial Pediatric Disease Medical Center, Kuming Medical University Affiliated Children’s Hospital, Kunming, Yunnan, China

**Keywords:** abdominal Henoch-Schönlein purpura, children, glucocorticoid, IgA vasculitis, matched cohort study, relapse, telitacicept

## Abstract

**Background:**

Severe abdominal Henoch-Schönlein purpura (HSP) can cause intense abdominal pain and gastrointestinal bleeding. Glucocorticoid therapy has limited efficacy and a high relapse rate after withdrawal. Telitacicept blocks BLyS/APRIL and inhibits the production of pathogenic IgA immune complexes, but its use in HSP has not been reported.

**Methods:**

This was a single-center retrospective matched cohort study. Children hospitalized with severe abdominal HSP (abdominal pain VAS ≥3, hematochezia, and extensive rash) between January 2025 and December 2025 were included. Patients were divided into a telitacicept plus glucocorticoid group (telitacicept group) and a glucocorticoid-alone group (steroid group). Individual 1:1 matching was performed based on age, sex, and disease severity. The primary outcome was time to abdominal pain relief. Secondary outcomes included time to rash resolution, disease relapse rate within 3 months after treatment, and safety. Intergroup comparisons used Wilcoxon rank-sum test or Fisher’s exact test, and relapse-free time was analyzed by Kaplan–Meier curves and the log-rank test.

**Results:**

A total of 26 children (13 per group) were included. Baseline characteristics were balanced between the two groups (age, sex, age strata, all P > 0.05). The median time to abdominal pain relief was 3.0 days (IQR 3.0–4.0) in the telitacicept group, significantly shorter than the 5.0 days (IQR 4.0–6.0) in the steroid group (P = 0.013). Median time to rash resolution was 9.0 days in both groups, with no significant difference (P = 0.113). The 3-month relapse rate was 7.7% (1/13) in the telitacicept group versus 46.2% (6/13) in the steroid group; the log-rank test showed a significant difference in relapse-time distribution (P = 0.022). The number needed to treat (NNT) was 2.6. For safety, mild injection-site redness occurred in 2 patients (15.4%) in the telitacicept group, and transient hyperglycemia occurred in 1 patient (7.7%) in the steroid group. No serious adverse events were observed.

**Conclusion:**

In children with severe abdominal HSP, adding telitacicept to glucocorticoid significantly shortens the time to abdominal pain relief and reduces the short-term relapse rate, with a favorable safety profile. This study provides preliminary clinical evidence for telitacicept as an adjunctive therapy to glucocorticoids, which warrants further validation in prospective randomized controlled trials.

## 1 Introduction

Henoch–Schönlein purpura (HSP) is the most common systemic small-vessel vasculitis in children, characterized by skin purpura, arthritis/arthralgia, abdominal pain, and renal involvement (1). Although most patients have a good prognosis, gastrointestinal involvement occurs in about 50–75% of cases, and 15–30% present with severe abdominal manifestations—intense abdominal pain, gastrointestinal bleeding, bowel wall edema, and even intussusception or bowel perforation (2). These children often require fasting, gastrointestinal decompression, intravenous fluid resuscitation, and antispasmodic/analgesic medications, leading to prolonged hospitalization, high medical costs, and a risk of glucocorticoid resistance or dependence.

Currently, glucocorticoids are first-line therapy for severe abdominal HSP. National and international guidelines recommend intravenous methylprednisolone pulse therapy or adequate oral prednisone, which generally controls acute symptoms (3). However, the limitations of glucocorticoids are equally prominent: (1) some children are insensitive to steroids, with delayed relief of abdominal pain (requiring addition of immunosuppressants such as cyclophosphamide or mycophenolate mofetil); (2) a high relapse rate during steroid tapering or withdrawal—literature reports a 3-month relapse rate of 30–60%, leading to repeated hospitalizations and cumulative steroid exposure; and (3) long-term steroid-related side effects including growth suppression, obesity, hyperglycemia, and osteoporosis (4, 5). Therefore, there is an urgent clinical need for an adjunctive therapy that can rapidly control symptoms, reduce relapse, and be safe.

Telitacicept is a recombinant TACI-Fc fusion protein that simultaneously blocks B-lymphocyte stimulator (BLyS) and a proliferation-inducing ligand (APRIL), thereby inhibiting plasma cell differentiation and antibody production (6). It has been approved in China for the treatment of systemic lupus erythematosus and has shown proteinuria reduction in a phase II clinical trial for IgA nephropathy (a disease whose pathogenesis is highly similar to that of HSP) (7). Theoretically, by reducing the formation of pathogenic IgA immune complexes, telitacicept might accelerate the resolution of inflammation in severe abdominal HSP and prevent relapse. However, no clinical data on telitacicept for severe abdominal HSP are currently available.

Therefore, we conducted this retrospective matched cohort study to preliminarily evaluate the efficacy and safety of telitacicept plus glucocorticoid versus glucocorticoid alone in children with severe abdominal HSP. The primary endpoint was time to abdominal pain relief, and secondary endpoints included time to rash resolution, 3-month relapse rate, and adverse events, aiming to provide preliminary evidence for future prospective randomized controlled trials.

## 2 Materials and methods

### 2.1 Study design and patient selection

This was a single-center retrospective matched cohort study. Patients hospitalized with severe abdominal Henoch–Schönlein purpura (HSP) at the Department of Pediatrics of our hospital between January 2025 and December 2025 were included. All cases met the 2019 European consensus meeting revision of HSP diagnostic criteria (3) and had the following severe abdominal manifestations: (1) severe abdominal pain (Visual Analog Scale [VAS] ≥3) with hematochezia; and (2) extensive skin purpura. Exclusion criteria were: use of glucocorticoids or immunosuppressants within 4 weeks before disease onset, concomitant joint symptoms or renal involvement (negative urine protein by dipstick, normal serum creatinine), active infection, or malignancy. Patients with joint symptoms or renal involvement at baseline were excluded from this study. This decision was made for two primary reasons. First, to establish a homogenous study population with severe abdominal involvement as the dominant clinical feature, thereby minimizing potential confounding effects of extra-abdominal manifestations (e.g., arthritis-related immobility or nephritis-related proteinuria) on the primary outcome, which was the time to abdominal pain relief. Second, as this is the first clinical evaluation of telitacicept in pediatric HSP, focusing on a well-defined, severe abdominal subgroup allows for a clearer and more direct assessment of the drug’s efficacy on the most urgent, life- and hospital-stay-impacting manifestation, providing a solid foundation for future investigations into its effects on other organ systems.

Patients were divided into two groups according to the treatment regimen: one group received telitacicept plus glucocorticoid (telitacicept group), and the other received glucocorticoid alone (steroid group). Among the steroid-group patients hospitalized during the same period, a 1:1 individual matching procedure was performed using a nearest-neighbor matching algorithm without replacement. The matching variables included age (± 2 years), sex, and disease severity. Disease severity was objectively quantified using the Visual Analog Scale (VAS) for abdominal pain (identical scores) and the presence of gross hematochezia (categorized as yes/no). Rash distribution was also matched (trunk and extremities). Laboratory parameters, such as white blood cell count, C-reactive protein, and serum creatinine, were not used as matching criteria but were compared between groups at baseline to ensure balance. Because this was a retrospective analysis, only previously collected clinical data were used, no treatment interventions were applied, and all data were de-identified before analysis. According to the “Measures for Ethical Review of Biomedical Research Involving Humans,” the study protocol was reviewed and approved by our hospital’s ethics committee, and the requirement for informed consent was waived (Ethics Approval No. 2025-03-401-K01).

### 2.2 Treatment regimens

Patients in the steroid group received intravenous methylprednisolone sodium succinate at a dose of 2 mg·kg^-^¹·d^-^¹, which was continued until marked relief of abdominal pain (score <3), then switched to oral prednisone (Tianjin Lisheng Pharmaceutical Co.) with gradual tapering, for a total course of 8–12 weeks. In the telitacicept group, based on the same glucocorticoid regimen, telitacicept (Remegen, China) was administered subcutaneously: 80 mg per dose for body weight ≥40 kg, or 1.5 mg/kg for body weight <40 kg, once weekly for 4 consecutive weeks. Both groups received conventional supportive care including proton pump inhibitors, fluid resuscitation, and electrolyte correction.

### 2.3 Data collection and outcome measures

The following data were extracted from the hospital’s electronic medical record system: demographic characteristics (age, sex), clinical manifestations (hematochezia, abdominal pain, rash, joint symptoms, renal involvement), medication records, and laboratory tests. The primary efficacy outcomes were: (1) time to abdominal pain relief—days from first drug administration to abdominal pain score <3 sustained for 24 hours; (2) time to rash resolution—days from first drug administration to no new rash and complete resolution of existing rash; (3) disease relapse within 3 months after treatment—defined as recurrence of abdominal pain (score ≥4) or new typical purpura. Follow-up time points were weeks 1, 2, 4, 6, 8, 12, and at the time of relapse. Two pediatric attending physicians independently assessed outcomes; disagreements were adjudicated by a senior physician.

### 2.4 Statistical analysis

Continuous variables were expressed as median (interquartile range) or mean ± standard deviation, and categorical variables as frequency (percentage). For intergroup comparisons, normality was checked using the Shapiro–Wilk test; normally distributed data were compared using the independent-samples t-test, non-normally distributed data using the Wilcoxon rank-sum test. Categorical variables were compared using Fisher’s exact test (when expected frequency <5) or Pearson’s χ^2^ test. Kaplan–Meier curves were used to plot relapse-free survival within 3 months after treatment, and intergroup differences were tested by the log-rank test. All tests were two-sided, with a significance level α = 0.05. Statistical analyses were performed using R 4.3.1 with the gtsummary, survival, and ggplot2 packages.

## 3 Results

### 3.1 Patient enrollment and baseline characteristics

By retrospective screening and 1:1 individual matching, a total of 26 children with severe abdominal HSP were included, 13 in each group. After matching, the two groups were highly balanced in age, sex, age strata, and clinical symptoms (**Table 1**). Median age was 9.0 years (range 5–15 years) in the telitacicept group and 9.0 years (range 5–14 years) in the steroid group; males accounted for 53.8% (7/13) in both groups. The distribution of age strata (<7 years, 7–12 years, >12 years) was 4 (30.8%), 5 (38.5%), and 4 (30.8%) in both groups. All patients presented with the triad of hematochezia, abdominal pain, and rash, and none had joint symptoms or renal involvement. Intergroup comparisons showed no significant difference in age distribution (Wilcoxon rank-sum test, P = 0.876), and no differences in sex or age-stratum composition (Fisher’s exact test, both P > 0.999). All patients in both groups were managed on the general pediatric ward; no patient required admission to the pediatric intensive care unit (PICU) at baseline, indicating a comparable level of initial care and disease severity between the groups.

**Table 1:** Baseline demographic and clinical characteristics of the matched cohort.

Variable	Overall N = 26^1^	Telitacicept N = 13^1^	Glucocorticoid N = 13^1^	p-value
Age (years)	9.0 (5.0-15.0)	9.0 (5.0-15.0)	9.0 (5.0-14.0)	0.876
Gender				>0.999
Female	12 (46%)	6 (46%)	6 (46%)	
Male	14 (54%)	7 (54%)	7 (54%)	
Age stratification				>0.999
<7 years	8 (31%)	4 (31%)	4 (31%)	
7–12 years	10 (38%)	5 (38%)	5 (38%)	
>12 years	8 (31%)	4 (31%)	4 (31%)	
Hematochezia				1
Yes	26 (100%)	13 (100%)	13 (100%)	
Abdominal pain				1
Yes	26 (100%)	13 (100%)	13 (100%)	
Rash				1
Yes	26 (100%)	13 (100%)	13 (100%)	
Joint symptoms				1
No	26 (100%)	13 (100%)	13 (100%)	
Renal involvement				1
No	26 (100%)	13 (100%)	13 (100%)	

1 Median (Q1-Q3); n (%).

2 Wilcoxon rank sum test; Fisher’s exact test.

Data are presented as median (first quartile – third quartile) for continuous variables and as number (percentage) for categorical variables. The telitacicept group (n=13) received telitacicept plus glucocorticoid, and the glucocorticoid group (n=13) received glucocorticoid alone. Groups were individually matched 1:1 by age (± 2 years), sex, severity of abdominal pain and hematochezia (identical scores), and rash distribution (trunk and extremities). Comparisons were performed using the Wilcoxon rank-sum test for age and Fisher’s exact test for all categorical variables. No statistically significant differences were observed between the two groups for any baseline variable (all P > 0.05), indicating successful matching. All patients presented with the triad of hematochezia, abdominal pain, and rash; none had joint symptoms or renal involvement at baseline. IQR, interquartile range.

### 3.2 Primary outcome: time to abdominal pain relief

The median time to abdominal pain relief was 3.0 days (interquartile range [IQR] 3.0–4.0) in the telitacicept group and 5.0 days (IQR 4.0–6.0) in the steroid group, a statistically significant difference (Wilcoxon rank-sum test, P = 0.013; **Figure 1A**). Using the steroid group as reference, the median difference in time to pain relief in the telitacicept group was −2.0 days (95% confidence interval [CI] −3.0 to −1.0). Using the Kaplan–Meier method to estimate cumulative relief rates (with pain not relieved as the event), the cumulative relief rate at day 4 was 76.9% in the telitacicept group versus only 23.1% in the steroid group. The log-rank test showed a highly significant difference in the distribution of time to pain relief between the two groups (χ^2^ = 6.82, P = 0.009) (**Figure 2**).

**Figure 1:**
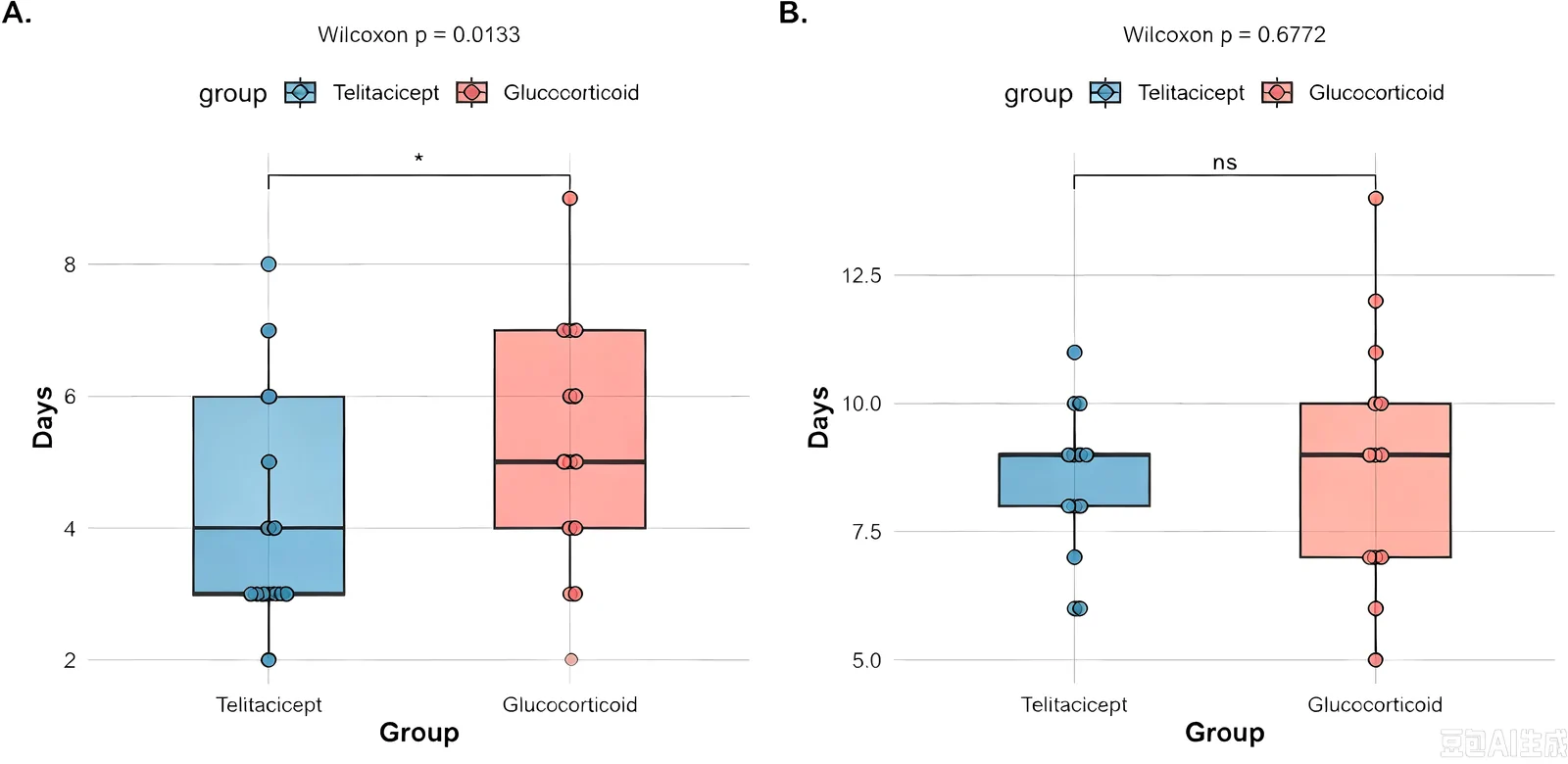
Comparison of time to abdominal pain relief and rash resolution (between the telitacicept plus glucocorticoid group and the glucocorticoid alone group. **(A)** Boxplots show the median (central line), interquartile range (box limits), and range (whiskers) of days to abdominal pain relief. Telitacicept group (n=13) had a median of 3.0 days (IQR 3.0–4.0), significantly shorter than the glucocorticoid group (n=13) with a median of 5.0 days (IQR 4.0–6.0). P = 0.013, Wilcoxon rank-sum test. **(B)** Boxplots of days to rash resolution show no statistically significant difference between the two groups: median 9.0 days for both, but with wider dispersion in the glucocorticoid group (IQR 7.0–10.0) than in the telitacicept group (IQR 8.0–9.0). P = 0.113, Wilcoxon rank-sum test. Each dot represents an individual patient.

**Figure 2:**
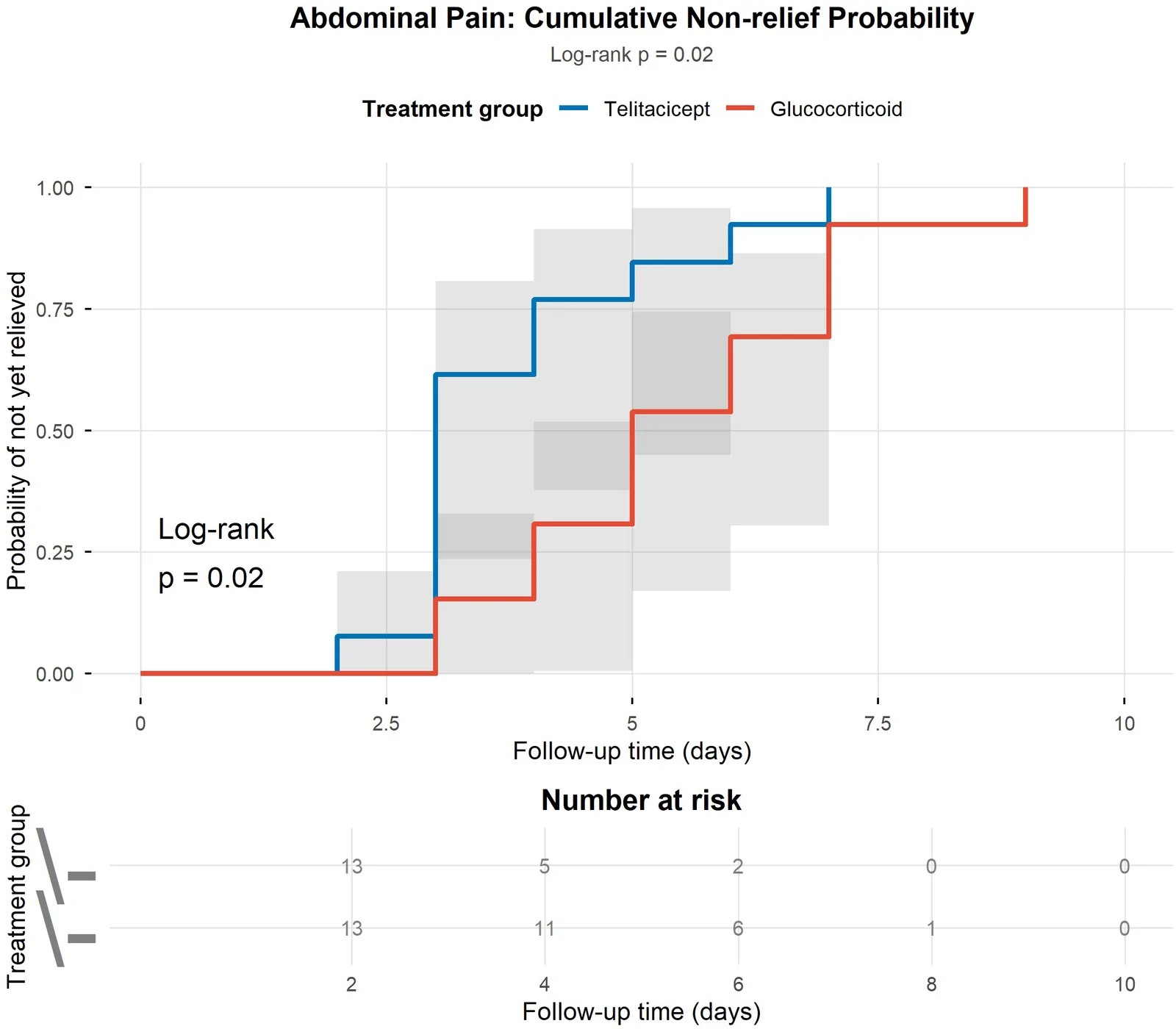
Kaplan–Meier curves for time to abdominal pain relief. Cumulative probability of remaining without abdominal pain relief is plotted against follow-up time (days). The telitacicept group (blue) achieved earlier pain relief compared with the glucocorticoid group (red). At day 4, the cumulative relief rate was 76.9% in the telitacicept group versus 23.1% in the glucocorticoid group. Log-rank test: χ^2^ = 6.82, P = 0.009. Censored patients (those who achieved relief) are indicated by tick marks on the curves.

### 3.3 Secondary outcome: time to rash resolution

The median time to rash resolution was 9.0 days (IQR 8.0–9.0) in the telitacicept group and 9.0 days (IQR 7.0–10.0) in the steroid group. The medians were identical, but the distribution in the steroid group was more dispersed (wider IQR). The Wilcoxon rank-sum test showed no statistically significant difference between the two groups (P = 0.113) (**Figure 1B**). Detailed distribution: the shortest time to rash resolution was 6 days and the longest 11 days in the telitacicept group; in the steroid group, the shortest was 5 days and the longest 14 days. In the steroid group, 4 patients (30.8%) had resolution beyond 10 days (one up to 14 days), whereas only 1 patient (7.7%) in the telitacicept group exceeded 10 days. Using day 7 as a cutoff, the rash resolution rate was 69.2% (9/13) in the telitacicept group versus 46.2% (6/13) in the steroid group, but Fisher’s exact test was not significant (P = 0.43); by day 9, the cumulative resolution rate was 84.6% (11/13) in the telitacicept group versus 69.2% (9/13) in the steroid group (P = 0.39) (**Figure 3**). Telitacicept did not show a clear advantage over glucocorticoid in accelerating rash resolution.

**Figure 3:**
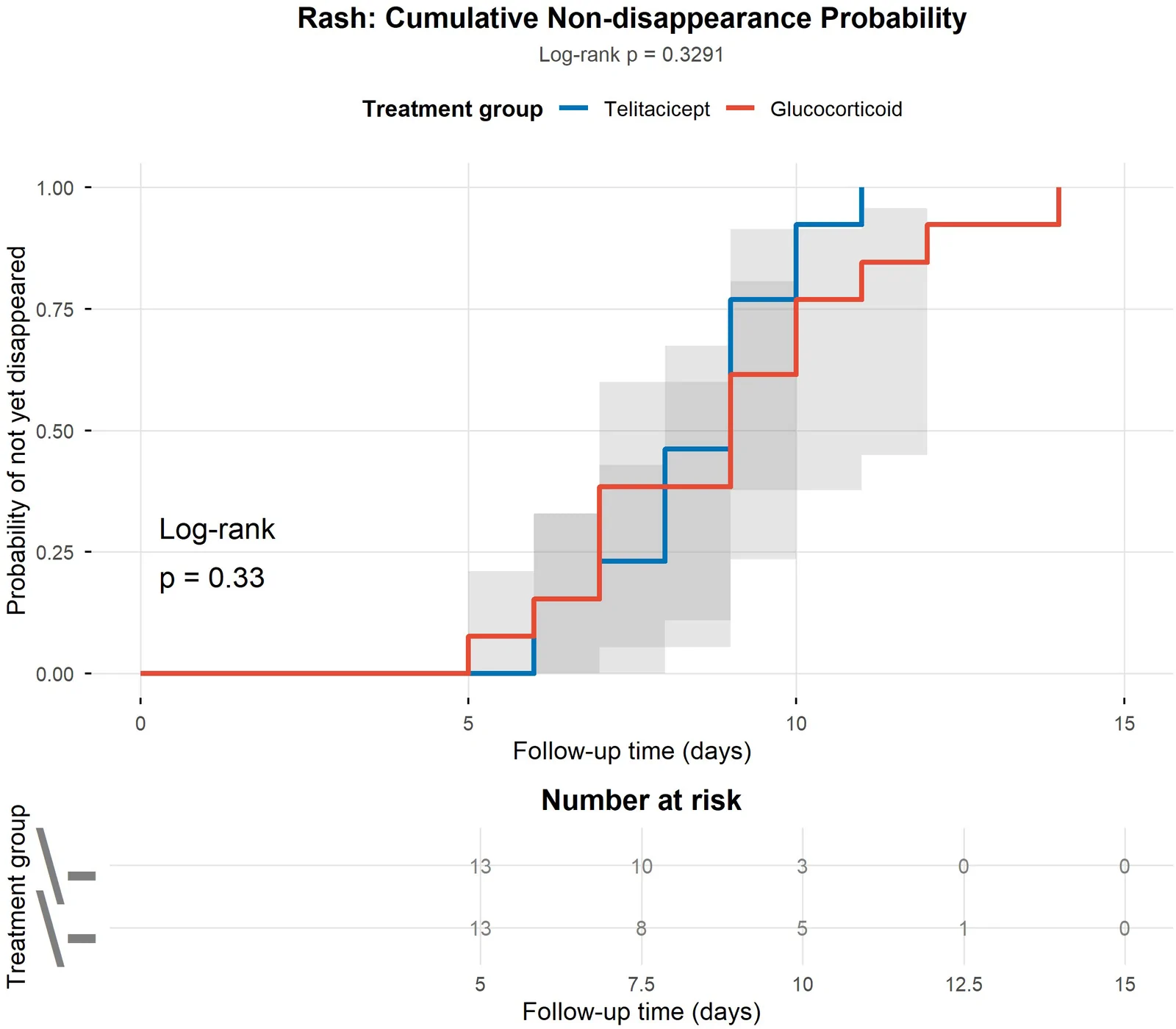
Kaplan–Meier curves for time to rash resolution. Cumulative probability of persistent rash (i.e., not yet resolved) over follow-up time (days). The telitacicept group (blue) showed a numerically higher resolution rate than the glucocorticoid group (red), but the difference was not statistically significant (log-rank P = 0.39). At day 9, cumulative resolution rates were 84.6% (11/13) in the telitacicept group and 69.2% (9/13) in the glucocorticoid group. Tick marks indicate censored patients (rash resolved).

### 3.4 Relapse outcome

During the 3-month follow-up after treatment, only 1 patient (7.7%) in the telitacicept group experienced disease relapse (mild abdominal pain, without hematochezia), whereas 6 patients (46.2%) in the steroid group relapsed, including 1 with recurrent abdominal pain, 4 with new purpura, and 1 with both. The Kaplan–Meier cumulative relapse probability curves (**Figure 4**) showed that the 3-month cumulative relapse rate was 7.7% (95% CI 1.1%–48.2%) in the telitacicept group and 46.2% (95% CI 23.2%–82.1%) in the steroid group. The steroid group reached a cumulative relapse rate plateau of 47% at 1.5 months of follow-up, whereas only one relapse occurred in the telitacicept group throughout the follow-up period. The log-rank test confirmed a significant difference in relapse-time distribution between the two groups (χ^2^ = 5.28, P = 0.022). The number needed to treat (NNT) was 2.6 (95% CI 1.6–8.9), meaning that treating 3 patients would prevent one relapse.

**Figure 4:**
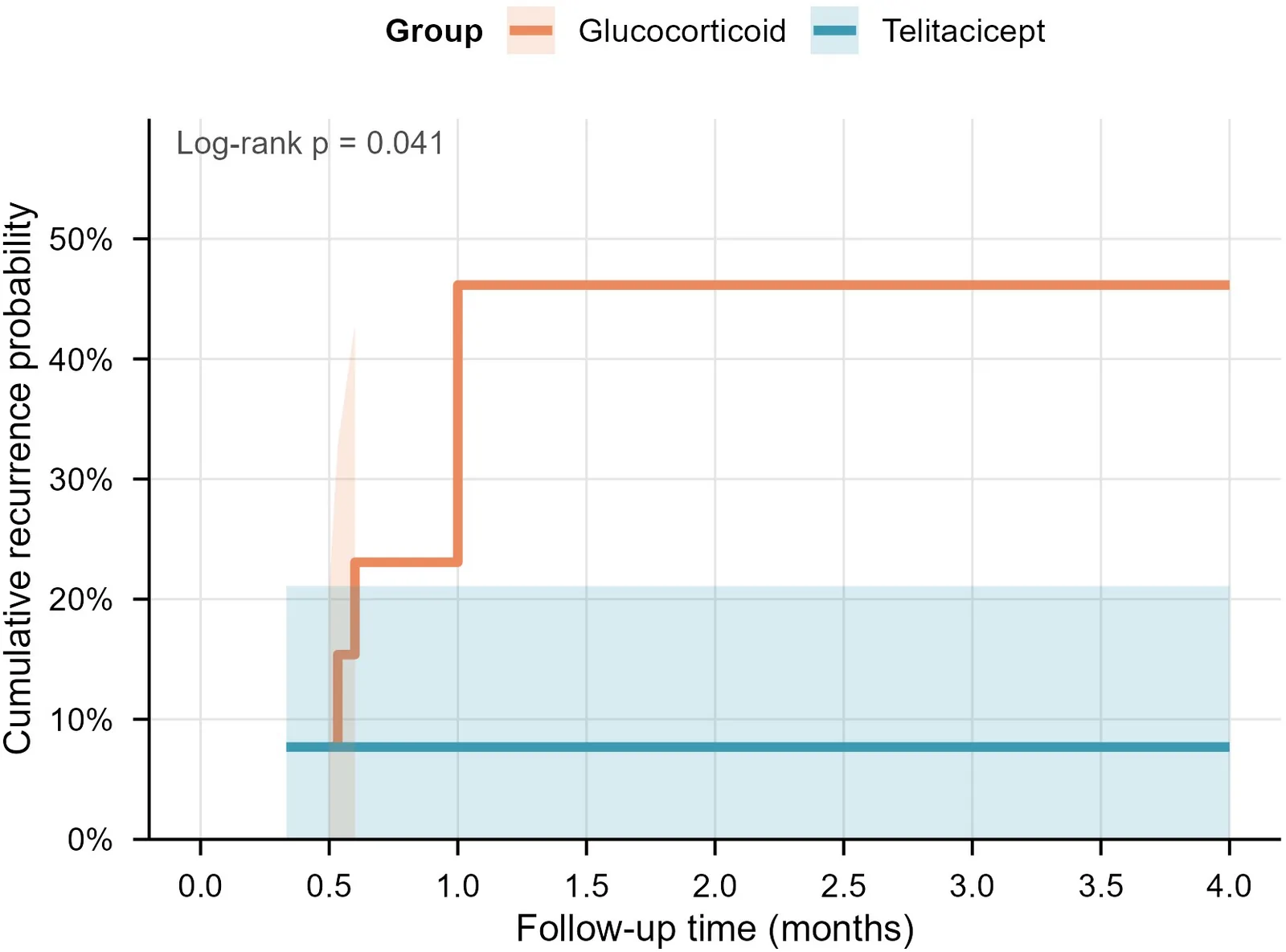
Kaplan–Meier curves for relapse-free survival within 3 months after treatment. Cumulative probability of relapse is shown as a function of follow-up time (months). The telitacicept group (blue) had a markedly lower relapse rate than the glucocorticoid group (red). At 3 months, cumulative relapse probability was 7.7% (95% CI 1.1%–48.2%) in the telitacicept group versus 46.2% (95% CI 23.2%–82.1%) in the glucocorticoid group. The glucocorticoid group reached a plateau of 47% at 1.5 months, whereas only one relapse occurred in the telitacicept group throughout the entire follow-up. Log-rank test: χ^2^ = 5.28, P = 0.022. Tick marks indicate censored patients (no relapse by the end of follow-up).

### 3.5 Safety

No serious adverse events (CTCAE grade ≥3) occurred in either group. In the telitacicept group, 2 patients (15.4%) developed mild redness at the injection site that resolved spontaneously within 24 hours without intervention. In the steroid group, 1 patient (7.7%) developed transient hyperglycemia (fasting blood glucose 7.8 mmol/L) that normalized after dietary control. No worsening of infection, gastrointestinal perforation, thromboembolic events, or deaths occurred in either group.

## 4 Discussion

In this study, we compared for the first time the efficacy and safety of telitacicept plus glucocorticoid versus glucocorticoid alone in children with severe abdominal Henoch–Schönlein purpura. The results showed that the combination therapy shortened the median time to abdominal pain relief from 5 days to 3 days (P = 0.013), reduced the relapse rate from 46.2% to 7.7% (P = 0.022), and although the difference in time to rash resolution was not statistically significant, the combination group showed a trend toward fewer prolonged cases. Both regimens were well tolerated, with no serious adverse events. These findings provide preliminary evidence for telitacicept as an adjunct to glucocorticoid therapy.

### 4.1 Clinical significance of abdominal pain relief

Abdominal pain is the most prominent symptom of abdominal HSP and the main reason for hospitalization. In this study, the median time to relief in the steroid-alone group (5 days) was consistent with previous literature (4–7 days), and adding telitacicept shortened it by 2 days. Considering that children require fasting, intravenous fluid resuscitation, and even antispasmodic/analgesic drugs until relief, a 2-day earlier relief not only reduces iatrogenic suffering but also shortens hospital stay and lowers medical costs. Notably, 76.9% of children in the telitacicept group achieved pain relief within 3 days versus only 23.1% in the steroid group. This early separation is particularly important for clinical decision-making—rapid symptom control helps enhance physician-patient confidence and avoids unnecessary glucocorticoid dose escalation or addition of second-line agents.

From a pathophysiological perspective, the abdominal manifestations of HSP result from deposition of IgA immune complexes in the small-vessel vasculature of the gastrointestinal tract, triggering leukocytoclastic vasculitis and tissue edema (8). Glucocorticoids primarily act by inhibiting the inflammatory cascade (reducing leukotrienes and prostaglandins) but have limited effect on upstream immune-complex formation and B-cell activation (9). Telitacicept, as a TACI-Fc fusion protein, simultaneously blocks BLyS and APRIL, thereby inhibiting the production of IgA by plasma cells (10). Theoretically, combination therapy might terminate the deposition of new immune complexes earlier, which could explain the accelerated relief of abdominal pain. However, as this is a hypothesis-generating study, further investigations with pharmacological and immunological assays are necessary to confirm this mechanism.

### 4.2 Why was there no benefit in time to rash resolution?

Unlike abdominal pain, the time to rash resolution did not differ significantly between the two groups (median 9 days in both). This may be due to several reasons. First, the inflammatory process in the skin vasculature differs from that in the gastrointestinal tract—clearance of immune complexes in the skin occurs primarily through the spleen and peripheral mononuclear phagocyte system (MPS) via Fc-receptor-mediated rapid elimination, rather than solely depending on blocking the formation of new complexes (11, 12). Second, glucocorticoids themselves promote resolution of existing rashes, so the “added value” of telitacicept may be diluted in cutaneous manifestations. Third, the assessment of rash resolution in this study had some subjectivity (definition of complete resolution), and the small sample size might have prevented detection of minor differences.

### 4.3 Significant reduction in relapse rate: potential mechanisms and clinical value

During the 3 months after treatment, the relapse rate in the telitacicept group was only 7.7%, much lower than the 46.2% in the steroid group. This difference (NNT = 2.6) is remarkable for a rare pediatric disease. We hypothesize that telitacicept, by inhibiting plasma cell survival and IgA production, may block the formation of “immunological memory” in HSP. Although traditional glucocorticoids control acute inflammation, immune reconstitution after withdrawal may lead to relapse; in contrast, the weekly dosing regimen of telitacicept maintains sustained B-cell modulation, keeping children in a state of low immune activity during glucocorticoid tapering. Notably, our definition of relapse included both recurrent abdominal pain and new purpura. Most relapses in the steroid group were purpura (4 isolated purpura relapses), whereas only one case in the telitacicept group was mild abdominal pain. Although purpura relapse is usually not life-threatening, it can cause patient anxiety and repeated medical visits, so the benefit of combination therapy is also meaningful in terms of quality of life.

### 4.4 Safety and comparison with existing evidence

No serious adverse events occurred in either group. The injection-site reaction rate (15.4%) for telitacicept was similar to that previously reported in children with lupus nephritis. Transient hyperglycemia occurred only in the steroid group, consistent with the known adverse effect profile. Because telitacicept was used for only 4 weeks (rather than long-term maintenance), the risk of infection was not increased. This is consistent with short-term safety data for telitacicept in adult IgA nephropathy (13). However, our cohort excluded patients with active infection or malignancy; in the real world, vigilance for opportunistic infections (especially reactivation of latent tuberculosis) is still required. There are currently no randomized controlled trials of telitacicept in HSP. Our results can be compared with a small case series (n = 8) using telitacicept for refractory HSP nephritis, which also reported good clinical response and tolerability.

### 4.5 Limitations

This study has several important limitations. First, although the retrospective matching design balanced baseline variables, unmeasured confounding (e.g., subtle differences in disease severity, prior medication history) might still exist. Second, the sample size was small (13 per group), leading to insufficient power for subgroup analyses; the negative result for time to rash resolution might be due to a type II error. Third, the follow-up period was only 3 months, so we could not assess long-term relapse risk or the impact of telitacicept on long-term renal outcomes (1–2% of HSP children develop IgA nephropathy years later). Fourth, and most importantly, the non-randomized treatment allocation inevitably introduces potential selection bias. In this real-world retrospective study, the decision to add telitacicept was at the discretion of the attending physicians and might have been influenced by unmeasured factors, such as a more refractory disease course perceived by the clinician, a family’s higher socioeconomic status allowing for more expensive biologics, or simply the parents’ preference for novel therapies. This indication bias could lead to an overestimation of the treatment effect. Although our matching strategy attempted to control for measured confounders, we cannot rule out residual confounding by these unmeasured variables. Therefore, our findings should be considered exploratory and hypothesis-generating, and require confirmation in a prospective, randomized controlled setting. Fifth, this was a single-center experience, so generalizability should be cautious. Based on these findings, we believe a multicenter, randomized, double-blind, placebo-controlled trial is warranted, with a primary endpoint of “time to complete abdominal pain relief” or “3-month cumulative relapse rate.” In addition, the optimal course of telitacicept (e.g., whether to continue for 4 weeks after glucocorticoid withdrawal) should be explored, and incorporation of biomarkers (e.g., serum IgA, galactose-deficient IgA1, B-cell subsets) would help elucidate the mechanism of action. Whether telitacicept has a renal protective effect in HSP children who already have renal involvement is also worth investigating. Specifically, the lack of these immunological correlates is a major limitation of our study, as our mechanistic speculation—that telitacicept accelerates immune-complex clearance—remains unsubstantiated by direct laboratory evidence. Future prospective studies are urgently needed to validate this hypothesis and to identify potential predictive biomarkers for treatment response.

## 5 Conclusions

In children with severe abdominal Henoch–Schönlein purpura, adding telitacicept to glucocorticoid therapy significantly shortens the time to abdominal pain relief and reduces the short-term relapse rate, with a favorable safety profile. Telitacicept may represent an effective adjunctive option to glucocorticoids for severe abdominal HSP.

## Data Availability

The original contributions presented in the study are included in the article/supplementary material. Further inquiries can be directed to the corresponding author.
